# Effects of increased milking frequency on gene expression in the bovine mammary gland

**DOI:** 10.1186/1471-2164-9-362

**Published:** 2008-07-31

**Authors:** Erin E Connor, Stephen Siferd, Theodore H Elsasser, Christina M Evock-Clover, Curtis P Van Tassell, Tad S Sonstegard, Violet M Fernandes, Anthony V Capuco

**Affiliations:** 1 Bovine Functional Genomics Laboratory, USDA-ARS, Beltsville Agricultural Research Center, 10300 Baltimore Avenue, Beltsville, MD 20705, USA; 2 Expression Analysis, Inc., Durham, NC 27713, USA

## Abstract

**Background:**

Previous research has demonstrated that increased milking frequency of dairy cattle during the first few weeks of lactation enhances milk yield, and that the effect persists throughout the entire lactation period. The specific mechanisms controlling this increase in milk production are unknown, but suggested pathways include increased mammary epithelial cell number, secretory capacity, and sensitivity to lactogenic hormones. We used serial analysis of gene expression (SAGE) and microarray analysis to identify changes in gene expression in the bovine mammary gland in response to 4× daily milking beginning at d 4 of lactation (IMF4) relative to glands milked 2× daily (Control) to gain insight into physiological changes occurring within the gland during more frequent milking.

**Results:**

Results indicated changes in gene expression related to cell proliferation and differentiation, extracellular matrix (ECM) remodeling, metabolism, nutrient transport, and immune function in IMF4 versus Control cows. In addition, pathways expected to promote neovascularization within the gland appeared to be up regulated in IMF4 cows. To validate this finding, immunolocalization of Von Willebrandt's factor (VWF), an endothelial cell marker, and its co-localization with the nuclear proliferation antigen Ki67 were evaluated in mammary tissue sections at approximately d 7 and d 14 of lactation in cows milked 4× daily versus Controls to estimate endothelial cell abundance and proliferation within the gland. Consistent with expression of genes related to neovascularization, both abundance of VWF and its co-localization with Ki67 appeared to be elevated in cows milked 4× daily, suggesting persistent increased milk yield in response to increased milking frequency may be mediated or complemented by enhanced mammary ECM remodeling and neovascularization within the gland.

**Conclusion:**

Additional study is needed to determine whether changes in ECM remodeling and neovascularization of the mammary gland result in increased milk yield during increased milking frequency, or occur in response to an increased demand for milk production. Gene pathways identified by the current study will provide a basis for future investigations to identify factors mediating the effects of milking frequency on milk yield.

## Background

It is well established that the frequency of milk removal from the mammary gland influences milk yield. Specifically, increased milking frequency in dairy cattle results in greater milk production [1,2], ranging from 10 to 15% [3]. It has been demonstrated that increased milking, when initiated during the early stages of lactation (first 6 wks) can enhance yield throughout the entire lactation period [4]. Furthermore, 4× daily milking for as short as the first 21 d in milk have resulted in persistent elevated milk yield [5-7]. The mechanisms contributing to this observed effect are unknown, but several factors have been suggested, including increased mammary epithelial cell number and differentiation [1,5], increased mammary cell secretory capacity [8], changes in apoptotic rate of mammary cells [5,9], and increased exposure [10] or sensitivity of the gland to prolactin [11]. Studies using half udder designs have indicated that enhanced milk production in glands milked 4× daily versus unilateral or contralateral glands milked 2× daily in early lactation is regulated locally within the gland, rather than by peripheral factors [1,7].

Another potential mechanism by which increased milking frequency may result in persistent enhancement of milk yield is through increased nutrient and lactogenic hormone delivery to the mammary gland via enhanced angiogenesis. Wall and McFadden [7] suggested that this mechanism is unlikely based on the acute decline in milk production they observed in response to a reduction from 4× to 2× daily milking, rather than a moderate decline one would expect with a change in vascularity. However, despite the initial decline in milk yield after cessation of 4× milking, the sustenance of increased milk yield into late lactation is consistent with a potential change in vascularity. Clearly, further study is needed to identify changes that occur within the mammary gland in response to increased milking frequency during early lactation that may contribute to increased milk yield and the persistence of such an increase.

High-throughput gene transcript profiling technologies, such as serial analysis of gene expression (SAGE) and microarray hybridization, are powerful approaches to evaluate global gene expression in tissues or cells of interest in response to experimental treatments or changes in physiological state. This information can provide insight into mechanisms contributing to observed physiological responses. For instance, the commercially available Affymetrix Bovine Genome Array has recently been applied to reproductive studies in cattle, including characterization of changes in gene expression that may be related to embryonic development [12], female fertility [13], and sperm development [14]. Likewise, SAGE has been used in multiple immunological studies of cattle to identify genes related to parasite resistance [15], antigen presentation in lymphocytes [16], and response to bovine viral diarrhea virus infection [17].

In the present study, SAGE and the Affymetrix Bovine Genome Array were used to characterize changes in expression of genes in the bovine mammary gland that may contribute to the increased milk production observed in response to an increase in milking frequency from 2× to 4× per day. Both approaches were used to get a more thorough assessment of differential gene expression and to evaluate the utility of SAGE to detect gene expression changes in the lactating mammary gland. Our results indicate that increased milking frequency alters expression of genes in the mammary gland related to increased extracellular matrix (ECM) remodeling, neovascularization, metabolism, cell proliferation and apoptosis. Factors identified by this work support our hypothesis that 4× daily milking increases the proportion of proliferating endothelial cells within the mammary gland and provide a basis for future studies to investigate specific mediators of enhanced milk production induced by increased milking frequency. The use of SAGE for transcript profiling in lactating mammary gland was deemed feasible; however, the high abundance of milk proteins, such as casein and β-lactoglobulin, make this approach less efficient due to the number of SAGE tags that are sequenced representing these proteins.

## Methods

### Animals and sample collection

The experimental design and sample collection are described in detail in Hale et al. [5]. Briefly, multiparous Holstein cows were milked either 2× daily (Control, n = 9) or 2× daily from d 1 to 3 of lactation then 4× daily beginning at d 4 of lactation until d 21 post partum (IMF4, n = 11). The milking intervals were approximately 12 h for Control cows and 9, 3, 9, and 3 h, respectively, for IMF4 cows. Milk somatic cell count (SCC) was measured weekly from two consecutive milkings using an infrared analyzer (Bentley Instruments, St. Paul, MN) during the first 10 wks of lactation. Mammary biopsies were obtained from a subset of cows at approximately d 7 and d 14 of lactation, processed and stored appropriately for subsequent analyses including RNA extraction and assessment of cell proliferation and apoptosis by immunohistochemical procedures. No biopsied mammary glands exhibited clinical signs of mastitis. Use of animals was approved by the Beltsville Agricultural Research Center's Animal Care and Use Committee.

To examine immediate changes in gene expression within the mammary gland in response to increased milking frequency, total RNA was isolated from d 7 mammary biopsy samples (n = 3 Control; n = 4 IMF4, mean ± SD actual sampling day = 6.3 ± 1.1) using the RNeasy Midi Kit (Qiagen, Valenica, CA). The IMF4 samples were selected for transcript profiling because significant changes in apoptotic percentage were observed in mammary epithelial cells of IMF4 cows at d 7 relative to Controls by Hale et al. (2003). Although additional animals were used in the study, sample sizes were limited to those of sufficient tissue quantity for RNA extraction. To remove contaminating genomic DNA, on-column DNase digestion was performed using the RNase-free DNase Set (Qiagen) according to kit instructions. RNA quality was assessed using the BioAnalyzer 2100 (Agilent Technologies, Palo Alto, CA; mean RIN = 7.1, range = 6.6 – 7.8) and concentrations were determined using a NanoDrop ND-1000 spectrophotometer (NanoDrop Technologies, Rockland, DE).

### SAGE library synthesis and analysis

Total RNA was pooled from equal quantities of control RNA samples (n = 3; 12 μg total) and IMF4 RNA samples (n = 4; 25 μg total) for SAGE library synthesis. Each SAGE library was constructed using the I-SAGE Long Kit (Invitrogen Corp., Carlsbad, CA) according to manufacturer's instructions. Plasmid clones from each library were purified using the Perfectprep Plasmid 96 Vac Direct Bind Kit (Eppendorf, Westbury, NY) and sequencing was conducted using an ABI 3730 automated DNA sequencer and BigDye (v. 1.1) chemistry (Applied Biosystems, Foster City, CA). Abundance of SAGE tags and differential expression was determined using SAGE2000 Version 4.5 analysis software (Invitrogen Corp.), with a normalization value of 12000 for "Compare" analysis. A minimum tag count of 4 in at least one of the two libraries was the minimum requirement for inclusion in the list of differentially expressed genes. Tags exhibiting <1.25-fold change in expression between treatment groups were also excluded from the list of differentially expressed genes. Identity of SAGE tag sequences was determined using the NCBI SAGEmap database [18].

### Microarray hybridization and data analysis

Microarray data were collected at Expression Analysis, Inc. (Durham, NC) using the GeneChip Bovine Genome Array (Affymetrix, Santa Clara, CA). These arrays contain probes for approximately 23,000 transcripts and variants, including over 19,500 UniGene clusters. The design of the array was based on content from Bovine UniGene Build 57 (March 24, 2004) and GenBank mRNAs.

Target was prepared and hybridized according to the "Affymetrix Technical Manual." Specifically, total RNA (2 μg) was converted to cDNA using reverse transcriptase (Invitrogen Corp.) and a modified oligo(dT)24 primer that contained T7 promoter sequences (GenSet, San Diego, CA). After first strand synthesis, residual RNA was degraded by addition of RNaseH, and a double-stranded cDNA molecule was generated using DNA polymerase I and DNA ligase. The cDNA was then purified and concentrated using a phenol:chloroform extraction, followed by ethanol precipitation. The cDNA products were incubated with T7 RNA polymerase and biotinylated ribonucleotides using the GeneChip IVT Labeling Kit (Affymetrix). The resultant cRNA product was purified using an RNeasy column (Qiagen) and quantified with a spectrophotometer. The cRNA target (20 μg) was incubated at 94°C for 35 min in fragmentation buffer (Tris, MgOAc, KOAc). The fragmented cRNA was diluted in hybridization buffer (MES, NaCl, EDTA, Tween 20, Herring Sperm DNA, Acetylated BSA) containing biotin-labeled OligoB2 and Eukaryotic Hybridization Controls (Affymetrix). The hybridization cocktail was denatured at 99°C for 5 min, incubated at 45°C for 5 min, and then injected into a GeneChip cartridge. The GeneChip array was incubated at 42°C for at least 16 h in a rotating oven at 60 rpm. GeneChips were washed with a series of nonstringent (25°C) and stringent (50°C) solutions containing variable amounts of MES, Tween20 and SSPE. The microarrays were then stained with streptavidin-phycoerythrin and the fluorescent signal was amplified by subsequently binding an anti-streptavidin antibody and streptavidin-phycoerythrin-labeled, biotinylated antibody. Fluorescent images were detected in a GeneChip Scanner 3000 and expression data was extracted using the GeneChip Operating System v 1.2 (Affymetrix).

An estimate of signal for each transcript was calculated using the Position-Dependent Nearest-Neighbor method [19] with a compression adjustment, and the results were analyzed via principal components analysis (PCA). Based on the PCA results, one of the IMF4 samples was identified as an outlier and removed prior to comparing the groups. Raw fold-change for each transcript was calculated by taking the simple ratio of the geometric means of the signal values for each respective group. Differential expression was determined using a robust implementation of permutation testing described on the Expression Analysis, Inc. website [20]. In brief, a modified t-statistic (D_*i*_) was calculated for each transcript when comparing groups, and a difference (Δ) was computed between D_*i *_and the average or expected t-statistic ordered values from a reference distribution (D_ [*i*]_) calculated by computing all possible random permutations of our samples. A list of differentially expressed transcripts was then created by first selecting any transcript with a suitable difference associated with a small false discovery rate (FDR), and further narrowed by selecting for transcripts with an estimated absolute raw fold change ≥1.25. Identity of transcripts was based on annotation by Affymetrix, Inc. [21] and by comparison of the target bovine UniGene sequence to the March 2005 Bovine Genome Sequence Assembly [22] using BLAT analysis. Gene functions were determined primarily using the NCBI Entrez Gene database [23], the MILANO annotation tool [24] and the Gene Ontology database [25].

### Validation of differential gene expression by quantitative real-time PCR

A subset of 11 genes (6 up-regulated and 5 down-regulated) identified as differentially expressed by microarray analysis and 4 genes (2 up-regulated and 2 down-regulated) differentially expressed as determined by SAGE were confirmed by absolute quantitative real-time PCR using the iCycler iQ Real-Time PCR Detection System (Bio-Rad Laboratories, Hercules, CA). Genes were selected to represent a broad range of differential expression levels as determined by fold change. Primer sequences for each gene target are presented in Table 1. Reverse transcription reactions to validate the 11 microarray-identified genes were conducted using the ABI High-capacity cDNA Archive Kit (Applied Biosystems, Foster City, CA) according to kit instructions using 6 μg of total RNA in a 60-μL reaction volume. Reaction conditions were 25°C for 10 min, followed by 37°C for 2 h. For the 4 SAGE-identified genes, reverse transcription was conducted using the iScript cDNA Synthesis Kit (Bio-Rad Laboratories) according to kit directions, using 750 ng of total RNA in a 30-uL reaction volume. Reaction conditions were 5 min at 25°C, 30 min at 42°C, and 5 min at 85°C. Negative control reactions were also performed on each RNA sample where reverse transcriptase was replaced with water in the reaction. Subsequent PCR was performed in duplicate (negative controls were performed in single reactions) using 2 μl of first-strand cDNA, 10 pmol of each primer and 12.5 ul of iQ SYBR Green Supermix (Bio-Rad Laboratories) in a 25-μl reaction volume, based on manufacturer's instructions. Cycling conditions consisted of 95°C for 3 min, 45 cycles of 94°C for 15 sec, annealing temperature for 30 sec, and 72°C for 30 sec, followed by a melting curve analysis. Annealing temperatures used were 56.8°C and 58.9°C for the 11 microarray-identified genes and 4 SAGE-identified genes, respectively. Standards (10^2 ^to 10^7 ^molecules) comprised of gel-purified PCR amplicons of each gene target were analyzed in duplicate for each assay, and a blank reaction using water as template was included with each standard curve. The iCycler Software was used to calculate amplification efficiency of each assay and transcript abundance of each unknown sample was extrapolated from corresponding assay standard curves. Transcript abundance was normalized to the quantity of input RNA in the reverse transcription reaction. Pearson correlation of fold-changes between the Control or IMF4 groups as determined by microarray and SAGE versus qPCR approaches for each gene was used to validate microarray results. Because fold change could not be calculated for *GCHFR *between the IMF4 and Control SAGE libraries due to the absence of tags for this gene in the control library (Control tags = 0, IMF4 tags = 9), a value of 9-fold was assigned for correlation analysis.

**Table 1:** Summary of gene targets evaluated by quantitative real-time PCR.

Gene	Amplicon size (bp)	Sense primer (5'→ 3')	Antisense primer (5'→ 3')
**Microarray-identified genes**			
*ALDH5A1*	116	ccttctctgcaggttgaagc	tcccacaaaatacagcatgaa
*CIDEA*	192	tcctacgacatccactgcac	cccctaccctctcttgatcc
*CTGF*	175	tgtgcagttggagcaacagt	cttccaggtggaaaaaccaa
*ECM1*	217	cgggatatcttgacccttga	agttacgtcgggaaccacac
*FGFR2*	168	ttggcgaatcttcatcacag	gtgagatcctgccagaggag
*IGFBP6*	161	aaggagagtaagccccaagc	agcacggagtccagatgttt
*MIA*	172	tgtttctcggtgtcgtcttg	atggtcaagaaacggcagtc
*PHLDA1*	131	gcaagtggactctggaccat	tacaatgatgcggaaggaca
*PTN*	215	gctgaccaagtccaaacctc	tcattttgtttctgcctattgtg
*SERPINF1*	245	gcctcagaaagtgacccaga	cgcgatgttccacttgagta
*SPADH1*	176	ctgagcacccagcttctttc	acaggctgagagcaggtgat
**Sage-identified genes**			
*GCHFR*	123	tggccttctctgaaacctgt	tttgacagaacagggccttc
*GLYCAM1*	115	caggcaaccacagagtcaga	tgcatcactgggagtgtgtt
*LTF*	107	tcggttattctggtgccttc	gtccctgtcagccttctctg
*LALBA*	125	ctctgctcctggtaggcatc	acagacccattcaggcaaac

### Immunohistochemistry

To validate whether increased milking frequency enhanced neovascularization in the mammary gland as suggested by our microarray results, mammary biopsy samples collected at d 7 and 14 of lactation from the 4 Controls and 4 IMF4 cows were evaluated for immunostaining of endothelial cells, using von Willebrand Factor (VWF) as a biomarker. In addition, mammary biopsies from 4 cows that were milked 4× daily beginning at d 1 of lactation (IMF1) were also evaluated by immunohistochemistry. Biopsies were fixed in 10% neutral buffered formalin overnight at 4°C and transferred to 70% ethanol. Samples were dehydrated and embedded in paraffin according to standard techniques and sectioned at 5 μm onto Superfrost™ plus slides (Erie Scientific Co., Portsmouth, NH). Slides were immunostained for simultaneous visualization of VWF and Ki67 antigens by brightfield microscopy, using the Picture™ Plus Double Staining Kit (Invitrogen Corp.). Antigen retrieval involved a 15-min incubation with trypsin (0.4 mg/ml in PBS containing CaCl_2_) as described by Robinson et al. [26] and microwave antigen retrieval in citrate buffer [27]. Endogenous peroxidase was blocked by 10-min incubation in 3% H_2_O_2 _in PBS and non-specific binding was blocked by incubating sections in CAS Block (Invitrogen Corp.) for 10 min at room temperature. The primary antibody (polyclonal rabbit anti-human VWF; 3.1 g/L; Dako North America Inc., Carpinteria, CA) was diluted 1:750 into a prediluted Ki67 primary antibody (monoclonal mouse anti-Ki67, prediluted, Invitrogen Corp.). Slides were incubated with both primary antibodies in a humidified chamber for 2 h at room temperature. Negative controls were performed using a non-immune rabbit and mouse IgG at appropriate concentrations. Slides were then co-incubated with goat anti-mouse IgG-horseradish peroxidase conjugate and a goat anti-rabbit IgG-alkaline phosphatase conjugate as the secondary antibodies for 30 min at room temperature. Secondary antibodies were visualized by staining with 3,3'-diaminobenzidine (DAB) for 4 min followed by fast red for 15 min. Sections were counter stained with Meyer's hematoxylin, washed, and mounted with Prolong Gold anti-fade reagent (Invitrogen Corp.; Prolong Gold proved an effective mounting medium for organic soluble chromogens such as fast red). Co-localization of VWF and Ki67 was indicative of a proliferating endothelial cell.

Abundance of endothelial cells in mammary sections was estimated by analyzing captured images of 12 randomly selected fields from four biopsy specimens per slide for each animal using an Olympus BX-40 microscope (10× objective) fitted with an Olympus DP-70 digital camera. Images were analyzed using the Image-Pro Plus Image Analysis Software (Version 4.5.1, MediaCybernetics Inc., Silver Spring, MD) through a standardized protocol previously described by Elsasser et al. [28]. Pixel counts for VWF were limited to only secretory epithelium and intralobular connective tissue (interlobular connective tissue was excluded) in each field. Pixel counts were constrained using the pre-filter and 8-connect object options. Counts were then normalized to the number of nuclei (blue) pixels, and normalized counts averaged by animal at a given biopsy period. Means were compared between treatments at each biopsy period using a t-test.

**Figure 1:**
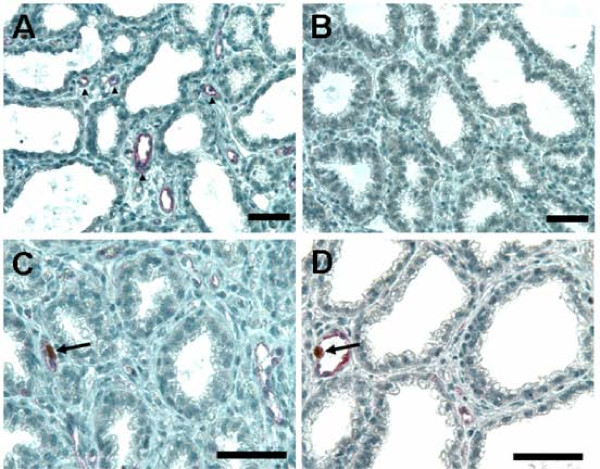
**Representative immunohistochemical detection of proliferating mammary endothelial cells**. **Panel A:** Endothelial cells were identified by staining for von Willebrand factor (VWF). Microvessels containing VWF-positive endothelial cells are evident and examples are indicated by arrows. **Panel B:** Negative control is depicted in which immune serum was replaced by non-immune isotypic control serum. **Panels C, D:** Examples of proliferative (Ki67-positive) endothelial cells are indicated by arrows. Magnification bar = 50 microns.

To quantify proliferating endothelial cells, tissue sections that were dually stained for VWF and Ki67 nuclear proliferation antigen were manually quantified. Figure 1 illustrates representative images showing immunohistochemical detection of proliferating mammary endothelial cells. Tissue sections were viewed at a magnification of 400× and non-overlapping fields were evaluated without knowledge of animal or treatment identification. The number of endothelial cells and endothelial cells that were Ki67-positive were recorded for mammary intralobular regions, whereas vessels in interlobular connective tissue were not evaluated. A minimum of 500 endothelial cells (VWF-positive cells) were evaluated per cow at each of the two biopsy time points (822 ± 58; mean ± SE). Presumed endothelial cells that were VWF-negative were not evaluated. The mean percentage of dually labeled cells was compared between treatment groups at each biopsy period using a t-test.

## Results

### Milk yield, mammary epithelial cell proliferation and apoptosis

A detailed description of the effects of increased milking frequency on milk yield, mammary cell proliferation, apoptosis, and other measures is described in Hale et al. [5]. Briefly, the IMF4 cows (n = 11) produced 4.8 kg/d more milk that the Control cows (n = 9) during the first 3 wks of lactation and 3.1 kg/d more milk during the complete 44-wk lactation (P < 0.05). Mammary cell proliferation on d 7 and d 14 as determined by ^3^H-thymidine incorporation was not different (P > 0.05) between the Control and IMF4 groups (n = 4/trt); however, immunohistochemical localization of Ki67 measured at d 14 was numerically greater in mammary epithelial cells of IMF4 cows compared to Control cows. In addition, the percentage of apoptotic mammary epithelial cells in IMF4 cows was greater than the percentage in Control cows (P < 0.05) sampled on d 7 of lactation. Similarly, the percentage of apoptotic stromal cells was greater than control cows on d 14 of lactation (P < 0.05). Milk components including fat and lactose percentage during the first 3 wks of lactation were also lower in IMF4 cows compared to Controls (P < 0.10).

### Differential gene expression determined by SAGE

A total of 27,432 tags were sequenced from the two SAGE libraries (14,548 IMF4 and 12,884 Control). Table 2 lists the differentially expressed SAGE tag sequences, their putative annotations and putative functions. Excluding the unique or unknown tags, all but three genes identified as differentially expressed by SAGE were represented on the microarray. As expected, the most abundant tags in both libraries were comprised of *α-S1-casein *(9%), *β-casein *(5–7%), *β-lactoglobulin *(4%), *κ-casein *(3%), *α-S2-casein *(2%), *α-lactalbumin *(1–2%) and *glycam 1 *(1%). Sixteen tag sequences were identified as up regulated in the IMF4 library, including 11 tags matching Unigene clusters, 2 tags matching no Unigene clusters but sampled from other bovine SAGE libraries (unknown), and 3 tags unique to our library (unique). Four tag sequences were present in the IMF4 library but absent in the Control library. A total of 28 tag sequences were identified as down regulated in the IMF4 library relative to the Control library, including 22 matching Unigene clusters, five unknowns, and one unique tag. Two of the tags matching Unigene clusters were unannotated genes. Ten of the down-regulated tags were absent in the IMF4 library but present in the Control library.

**Table 2:** Changes in mammary gene expression in response to four times daily milking^1 ^(IMF4) as determined by serial analysis of gene expression (SAGE).

		Number of tags				
					
Tag	Gene symbol	Control	IMF4	P-value	UniGene annotation	Putative function
**Genes up regulated in IMF4**						
GCTCCAGGAC	*GCHFR*	0	9	0.0034	*GTP cyclohydrolase I feedback regulator*	regulation of NO synthesis
GACGACACGA	*RPS28*	0	6	0.0214	*ribosomal protein S28*	protein biosynthesis
CAGCAAGGTG	*STK19*	0	5	0.0385	*serine/threonine kinase 19*	transcriptional regulation
TCTCCCCTGT	*GAK*	0	5	0.0385	*cyclin G associated kinase*	membrane trafficking, putative tumor suppressor
AGGAGTTGGG		1	14	0.001	*major histocompatibility complex, class I, A*	immune response
GGCCTGATCG	*LTF*	2	17	0.0009	*lactotransferrin*	defense against bacterial infection
GAAATCATTG	*UGP2*	2	10	0.0304	*UDP-glucose pyrophosphorylase 2*	lactose synthesis
CCGGTATAAA		4	15	0.0214	unique^2^	
CCGGTCGCCC	*RPS9*	8	23	0.0124	*ribosomal protein S9*	protein biosynthesis
GCAGTGGTAG		8	23	0.0124	unknown^3^	
GCGGCCAGCG	*LGB*	12	32	0.0064	*β-lactoglobulin*	component of whey protein
CAGATATAGG		15	31	0.0351	unknown	
GCCGGCCCGG	*RPS15*	23	44	0.0253	*ribosomal protein S15*	protein biosynthesis
GGCAGGGCCT	*H2AFY2*	48	87	0.0045	*H2A histone family, member Y2*	transcriptional regulation
CAGAAGAGCA		24	42	0.0481	unique	
ATACTACTGG		70	120	0.0033	unique	
**Genes down regulated in IMF4**						
CCTTTGAAGT	*GLYCAM1*	117	92	0.0055	*glycosylation dependent cell adhesion molecule 1*	cell adhesion molecule
TCCATTGTCA		67	51	0.0185	unknown	
GTAGTTGGCT		106	80	0.0037	unique	
AACATATCAA	*CSN2*	894	574	0	*β-casein*	defense against infection
GGAAAGGCGG	*MFGE8*	29	18	0.0303	*milk fat globule-EGF factor 8 protein (lactadherin)*	remodeling during mammary involution, neovascularization
AATGGCGCTC	*LALBA*	195	120	0	*α-lactalbumin*	lactose synthesis, antimicrobial and anti-tumor function in neonate
GGTTCAAAAC		20	10	0.0235	unknown	
GCTGAATAAA	*MUC1*	13	6	0.0486	*mucin 1, transmembrane*	defense against microbial infection, cell signaling
GAATAGGATG		21	8	0.0045	unknown	
CACCCCTGGA		11	4	0.0379	unknown	
CAAACTCTTG	*ATOX1*	11	3	0.0163	*antioxidant protein 1*	ion transport, protective role in oxidative stress
AAAATAAAAG	*CXCL3*	8	2	0.0405	*chemokine (C-X-C motif) ligand 3*	immune response
ACCCTGTGCC		9	2	0.0216	*LOC506990*	
ACCAAAAACC	*COL1A1*	27	5	0	*type I collagen, α-1*	component of extracellular matrix
ACCCGGCTGA		6	1	0.0406	*MGC127561*	
ATCAATAAAG	*CAPG*	7	1	0.0229	*capping protein (actin filament), gelsolin-like*	tumor suppressor, control of cellular motility
CTCGATGAAA	*XBP1*	8	1	0.0135	*X-box binding protein 1*	immune response
GCCCACAAAG	*FGFBP1*	9	1	0.0065	*fibroblast growth factor binding protein 1*	epithelial cell repair after injury, angiogenesis
GCGCCCCTGC		10	0	0.0006	*major histocompatibility complex, class I, A*	immune response
CACACCCGGC	*MRPL45*	8	0	0.0024	*mitochondrial ribosomal protein L45*	protein biosynthesis
GTCTTTAATT		8	0	0.0024	unknown	
CTTTGTGTGC	*ALDOC*	5	0	0.0225	*aldolase C, fructose-bisphosphate*	cellular metabolism
GGCCCAGTTT	*PPIB*	5	0	0.0225	*cyclophilin B*	chaperone facilitating action of lactogenic hormones, inflammatory response
CAGATTTGTG	*DDX24*	4	0	0.0467	*DEAD (Asp-Glu-Ala-Asp) box polypeptide 24*	RNA helicase
CCCGCGGAAA	*SAA3P*	4	0	0.0467	*serum amyloid A3*	defense against bacterial infection, remodeling of mammary gland
GCCGAAGCTA	*TSC2*	4	0	0.0467	*tuberous sclerosis 2*	inhibitor of cell growth and proliferation
TGAGGCTCAC	*QDPR*	4	0	0.0467	*quinoid dihydropteridine reductase*	L-phenylalanine catabolism
TTTGGTTTTC	*COL1A2*	4	0	0.0467	*type I collagen,α-2*	component of extracellular matrix
GCTCCAGGAC	*GCHFR*	0	9	0.0034	*GTP cyclohydrolase I feedback regulator*	regulation of NO synthesis
GACGACACGA	*RPS28*	0	6	0.0214	*ribosomal protein S28*	protein biosynthesis
CAGCAAGGTG	*STK19*	0	5	0.0385	*serine/threonine kinase 19*	transcriptional regulation
TCTCCCCTGT	*GAK*	0	5	0.0385	*cyclin G associated kinase*	membrane trafficking, putative tumor suppressor
AGGAGTTGGG		1	14	0.001	*major histocompatibility complex, class I, A*	immune response
GGCCTGATCG	*LTF*	2	17	0.0009	*lactotransferrin*	defense against bacterial infection
GAAATCATTG	*UGP2*	2	10	0.0304	*UDP-glucose pyrophosphorylase 2*	lactose synthesis
CCGGTATAAA		4	15	0.0214	unique^2^	

^1 ^Cows were milked twice daily from d 1 to 3 of lactation, and four times daily beginning at d 4 of lactation until d 21 post partum

^2 ^Tag sequence is unique to this SAGE library

^3 ^Tag sequence matches no Unigene clusters but has been sampled from other bovine SAGE libraries

Consistent with an increase in milk yield, approximately half of the annotated tags that were up regulated in the IMF4 library represented genes functioning in protein biosynthesis and transcriptional regulation. One gene, *GTP cyclohydrolase I feedback regulator *(*GCHFR*) functions in the down regulation of endothelial cell nitric oxide synthesis and plays a role in immune response and vasoconstriction. Likewise, transcripts for the immune-related protein lactotransferrin were up regulated in the IMF4 group.

Genes that were down regulated by increased milking frequency included those functioning in defense against microbial infection or immune response (8 out of 20 annotated genes), remodeling of the mammary gland, regulators of angiogenesis, or as constituents of the ECM. In addition, *tuberous sclerosis 2 *(*TSC2*), an enhancer of TGF-β signaling [29] and inhibitor of cell proliferation and differentiation was down regulated in IMF4 cows, as well as the tumor suppressor *CAPG*. Multiple SAGE tags representing the classical *Major Histocompatibility Complex (MHC) class I antigen (BOLA) *gene locus were sampled, of which one tag was identified as up regulated and the other was identified as down regulated by increased milking frequency. Likewise, this gene locus was represented on the microarray by multiple probes, which were both up and down regulated in the IMF4 cows. Three genes (*ATOX1*, *COL1A1*, and *COL1A2*) identified as down regulated in the IMF4 library were also identified as down regulated by microarray analysis. However, *RPS28 *was identified as up regulated in the IMF4 library by SAGE, but down regulated in IMF4 cows by microarray analysis. In addition, expression of *α-lactalbumin*, a key regulatory enzyme for lactose synthesis was lower in IMF4 cows than in Control cows, consistent with the lower percentage of lactose measured in IMF4 cows during this period.

### Differential gene expression determined by microarray

A total of 183 genes were identified as differentially expressed by microarray analysis, including 27 unknown transcripts [see Additional file 1]. Compared to Control cows, 64 genes were up regulated and 117 were down regulated in IMF4 cows. Two genes (*BLA-DQB *and *BOLA*) were identified as both up and down regulated by microarray, depending on the probe analyzed.

Eighteen differentially expressed genes were represented by multiple probes on the microarray. For these genes, the direction of fold change was consistent among all probes, with the exception of *BLA-DQB *and *BOLA *[see Additional file 1]. Expression of multiple members of several gene families were also identified as responsive to increased milking frequency including: the CIDE family of cell death activators (*CIDEA *and *CIDEC*), collagens (*COL1A1*, *COL1A2*, *COL3A1*, *COL5A2*, *COL6A1*, *COL6A3*, and *COL12A1*), fibrinogens (*FGG *and *FGL2*), heat shock proteins (*HSPB6 *and *HSPB8*), microfibril-associated proteins (*MFAP2 *and *MFAP5*), ras and ras-like proteins (*RASL11B*, *RASSF4*, *DIRAS3*, *RASL11B *and *HRASLS3*), ribosomal proteins (*RPL37A*, *RPL38*, *RPS21 *and *RPS28*), S100 calcium binding proteins (*S100A4 *and *S100A10*), serine proteinase inhibitors (*SERPINF *and *SERPING1*), secreted frizzled-related proteins (*SFRP2 *and *SFRP4*), solute carriers (*SLC1A5*, *SLC39A8 *and *SLC7A5*), thrombospondins (*THBS2 *and *THBS4*), tenascins (*TNC *and *TNXB*), and tissue inhibitors of metalloproteinases (*TIMP1 *and *TIMP2*).

**Figure 2:**
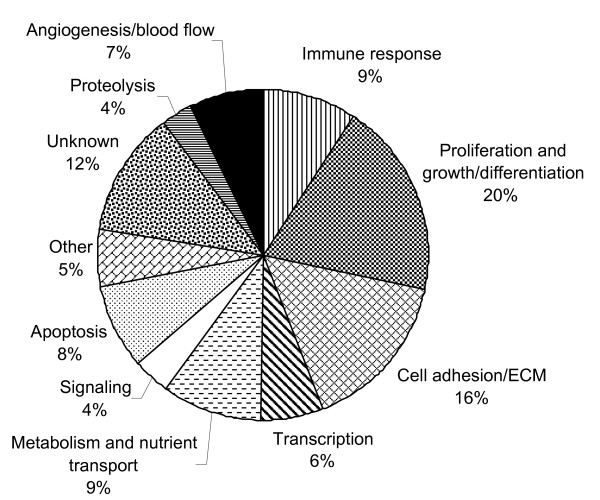
**Functional categorization of genes responsive to milking frequency in the mammary gland**. Transcripts that were up and down regulated are depicted. Milking frequency affected expression of 183 genes, including 27 transcripts whose identity is unknown.

As shown in Figure 2, the majority of genes influenced by milking frequency whose functions have been investigated included: genes controlling cell proliferation and growth or differentiation (20%); components of the ECM or functioning in cell adhesion (16%); genes involved in metabolism or nutrient transport (9%); and genes participating in immune response (9%).

#### 1. Changes in regulators of cell proliferation and differentiation

A total of 53 genes were identified that regulate progression through cell cycle, cell proliferation, cell differentiation, chromatin remodeling, or are known tumor suppressors or oncogenes. For instance, expression of the putative tumor suppressors *BANP*, *DAB2*, *DIRAS3*, *FAT2*, *HRASLS3*, *LOX*, *PDGFRL *and *RASSF4 *and the putative oncogene *BCAS1 *was altered by increased milking frequency. Known growth factors and regulators of cell cycle progression were also differentially expressed in mammary glands of IMF4 and Control cows, such as *insulin-like growth factor-binding protein 6 *(*IGFBP6*), *connective tissue growth factor *(*CTGF*), *Kruppel-like factor 11 *(*KLF11*), *fibroblast growth factor receptor 2 *(*FGFR2*), *platelet-derived growth factor C precursor *(*PDGFC*), and *S100 calcium-bindingproteins A10 *and *A4 *(*S100A10 *and *S100A4*). Likewise, factors regulating Wnt signaling (*SFRP2*, *SFRP4*, and *TCF7L2*), and differentiation of neurons (*CHRDL1*, *NDRG2*, *NRG1*, and *NTRK2*) and muscle (*FHL1*) were altered by milking frequency.

Multiple genes controlling proliferation of blood vessels were affected by increased milking frequency including down regulation of inhibitors of angiogenesis such as *SERPINF1*, *THBS2*, *TIMP1*, *TIMP2 *and *ADIPOQ *[30], and up regulation of pro-angiogenic genes *CTGF*, *NRG1 *and *ANTXR1*. Down regulation of several genes that promote angiogenesis (*AGTR1*, *EGR1*, *NELL2*, *S100A4*, *ANGPTL2 *and *F2R*) was also observed in IMF4 cows.

#### 2. Changes related to ECM remodeling

Expression of nearly 50 genes encoding ECM components or structural elements of the cytoskeleton, participating in cell adhesion, and in regulation of proteolysis was altered by milking frequency. Nearly all of these genes were down-regulated in IMF4 cows and indicated remodeling of ECM and changes in cell adhesion. For instance, only *FAT2*, a member of the cadherin family, *PIK3R1*, an inhibitor of cell adhesion, and *CTGF *were up regulated by increased milking frequency. Genes encoding components of ECM including numerous collagens, versican, elastin, fibulin, fibronectin 1, microfibrillar associated glycoproteins, thrombospondins, myocilin, tenascins, and lumican were down-regulated in mammary gland of IMF4 cows. Furthermore, known regulators of ECM deposition and remodeling (e.g. *S100A4*, *LOX*, *PCOLCE*, *TIMP1 *and *TIMP2*) and proteolysis (e.g. *BACE2*, *CTSK*, *FAP *and *UBD*) were down regulated in IMF4 cows.

#### 3. Changes in metabolism and nutrient transport

Differential expression of a number of genes related to metabolism and nutrient transport to support milk secretion was observed in response to milking frequency. Specifically, transporters involved in vesicular protein trafficking (*TMED10*) and transport of nutrients such as *aquaporin 5 *(*AQP5*), several solute carriers (*SLC7A5*, *SLC39A8 *and *SLC1A5*) and the fatty acid transporter *carnitine O-octanoyltransferase *(*CROT*) were up regulated in mammary gland of IMF4 cows, whereas genes involved in phosphate transport (*COL12A1*, *SCARA5 *and *SLC34A2*) and copper transport (*ATOX1*) were down regulated in IMF4 versus Control cows. Lastly, expression of multiple metabolism-related genes were altered by increased milking including the increased expression of genes controlling catabolism of UDP (*ENTPD4*), glycoproteins (*AGA*) and fatty acids (*CPT1A*), and biosynthesis of cholesterol (*SC4MOL*), isoprenoids (*IDI1*) and prostaglandins (*AKR1C1*). Increased milking frequency also suppressed expression of *ADIPOQ*, an inhibitor of gluconeogenesis, and isocitrate dehydrogenase (*IDH1*), which functions in fatty acid synthesis.

#### 4. Changes in immune-related genes

Increased milking frequency affected expression of several genes that function in immune response and inflammation. For instance, multiple genes within the *MHC *loci (*BOLA*, *BLA-DQB*, *BOLA-DRB3*, *BOLA-DQA1*, *LOC510417*, *UBD *and *CFB*) were differentially expressed in mammary gland of IMF4 cows compared to Control cows. Furthermore, genes related to innate immunity including chemokines *CCL26 *and *LOC504773*, the complement pathway regulator *SERPING1 *and pathway member *CFB*, the interferon-induced genes *ISG12(A) *and *FGL2*, and the proinflammatory molecule *FSTL1 *were down regulated. Up regulated genes included those involved in acute-phase response, such as *FGG *and *IL1RAP*, and a regulator of complement activation, *HF1*.

### Validation of differential gene expression by quantitative real-time PCR

**Figure 3:**
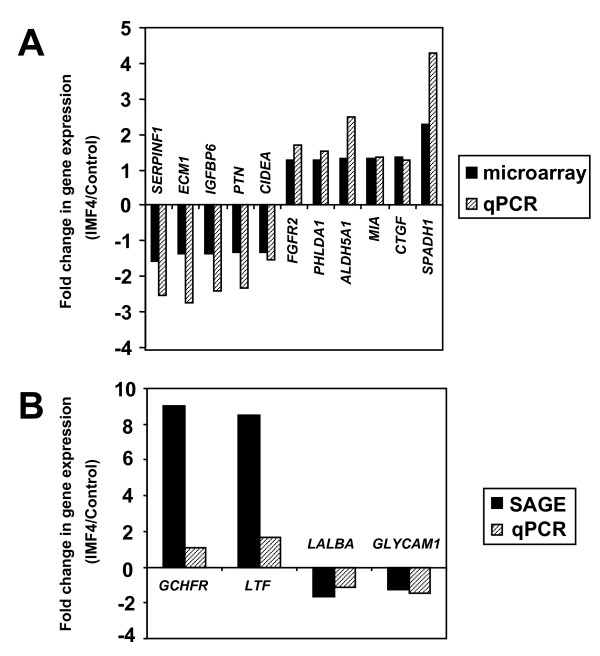
**Validation of differential gene expression by quantitative real-time PCR**. Relationships between the gene expression levels in cows milked 4× daily (IMF4) relative to cows milked twice daily (Control) as determined by quantitative real-time PCR (qPCR) versus microarray and SAGE approaches are illustrated. **Panel A:** Fold changes as determined by microarray versus qPCR (r = 0.97; P < 0.00001); **Panel B:** Fold changes as determined by SAGE versus qPCR (r = 0.98; P = 0.023).

Four genes identified by SAGE and eleven genes selected to represent a range of differential expression among the up- (1.26- to 2.27-fold) and down-regulated (-1.32- to -1.57-fold) genes identified by microarray were validated by real-time PCR. Amplification efficiency of assays ranged from 81 to 107% and correlation coefficients of all standard curves were ≥0.997. In all cases, the direction of change was confirmed and the correlation of the estimated fold-change between the two methods was extremely high (r = 0.97, P < 0.00001 [microarray]; r = 0.98; P = 0.023 [SAGE]; Figure 3).

### Immunohistochemistry

Consistent with microarray results, normalized pixel abundance for VWF, a biomarker for endothelial cells and vascularity, was measured in sections of d-7 biopsies and was numerically greater in IMF4 cows (2.46 ± 0.80; n = 4) than in Control cows (1.83 ± 0.45; n = 4) (P = 0.21). Combining results of IMF1 cows with IMF4 cows in the analysis further improved this trend (mean of IMF = 3.28 ± 1.06; n = 7) (P = 0.12). However, immunostaining for VWF in mammary biopsies collected at d 14 of lactation was similar between IMF cows and Controls (data not shown).

Co-localization of VWF with Ki67 in d-7 biopsies indicated the percentage of dually labeled cells in IMF4 cows (n = 4) was numerically greater than in Controls (n = 4) at 1.03 ± 0.52% versus 0.73 ± 0.15%, respectively (P = 0.31). Inclusion of IMF1 cows (n = 4) with the IMF4 data set also improved this trend (mean of IMF = 1.12 ± 0.33%; P = 0.16). Measures of d-14 biopsies indicated a similar and slightly stronger relationship of 1.07 ± 0.37% versus 0.66 ± 0.17% for IMF4 and Control groups, respectively (P = 0.19). Finally, combining all IMF animals (n = 8) resulted in an even greater increase in mean percentage of VWF/Ki67 co-localization relative to Controls (1.21 ± 0.31%; P = 0.07).

## Discussion

Increased milking frequency of 4× daily milking versus 2× daily milking in early lactation of dairy cattle results in a persistent increase in milk yield, well beyond the period of increased milking [5,7,10]. Changes occurring within the mammary gland that may facilitate the increased milk production include increased mammary cell proliferation and differentiation [1,5,10]. In the current study, the greatest percentage of genes affected by milking frequency, as determined by microarray, functions in these processes. Although changes in gene expression related to cell proliferation in IMF4 cows were not unidirectional (i.e., consistently promoting proliferation), one would expect to see feedback regulation of genes controlling cell proliferation in order to maintain tissue homeostasis. Furthermore, an increase (P < 0.05) in ^3^H-thymidine incorporation at 7 d in mammary tissue of IMF1 cows and a numerical increase in Ki67 labeling of epithelial cells in the glands of IMF4 cows at d 14 of lactation in the present study [5] supports the hypothesis that changes in gene expression were occurring in the gland that promote mammary epithelial cell proliferation.

Changes within the mammary gland related to endothelial cell proliferation were also indicated in response to treatment. In order to support greater milk production, increases in neovascularization and blood flow would be expected within the gland to enhance nutrient delivery to mammary secretory cells. Evidence of genetic changes related to angiogenic responses included increased expression of stimulators of blood vessel development and suppression of inhibitors of angiogenesis in IMF4 cows. Because angiogenesis is a tightly regulated process, it is not surprising that down regulation of stimulators of angiogenesis were also observed with the mammary glands of IMF4 cows. In fact, autocrine regulation of angiogenesis through production of anti-angiogenic factors by endothelial cells is a means by which the balance between growth activation and suppression is maintained [31].

In addition to changes in expression of genes directly affecting blood vessel development, changes in expression of genes promoting ECM remodeling were also indicated in response to increased milking frequency. It has been shown that the ECM is critical in regulating neovascularization and endothelial cell survival [31,32] and that proteolysis and remodeling of the ECM are necessary to allow for the invasion of new blood vessels [33]. Therefore, we speculated that changes were occurring within the ECM in response to increased milking that promote neovascularization.

Immunolocalization of VWF in mammary sections suggested greater vascularization at approximately d 7 in IMF4 cows relative to Control cows, consistent with our microarray data implicating changes in expression of genes that are relevant to neovascularization. To determine whether proliferation of endothelial cells was altered within the gland by milking frequency, we also examined the co-localization of the endothelial cell marker VWF and Ki67 by immunohistsochemical analysis of mammary sections collected on d 7 and d 14 of lactation. Our results, although not statistically significant, showed consistently numerically greater co-localization in IMF cows, which supports our hypothesis that 4× daily milking increased the proportion of proliferating endothelial cells. Experiments specifically designed to test this hypothesis using greater numbers of animals and frequent sampling times are warranted. Furthermore, additional study is needed to determine whether changes in ECM remodeling and neovascularization precede or lag increases in milk yield to ascertain whether these changes serve as a primary mechanism for the enhancement of milk production, or occur as a result of the increased demand for milk synthesis. Of interest, research by Lacasse and Prosser [34] suggests that increases in mammary blood flow do not result in increases in milk production in lactating goats. Even though neovascularization may not be a driving force behind increased milk production during frequent milking, the persistence of increased tissue perfusion may account for the persistence of increased milk production after conclusion of the period of frequent milking.

Physiological mechanisms resulting in increased milk yield during more frequent milking may also include increases in renewal of mammary epithelial cells via apoptosis. Hale et al. [5] reported an increase in the percentage of apoptotic mammary cells in IMF4 cows from this study as determined by in situ labeling of apoptotic cells using terminal uridine deoxynucleotidyl transferase dUTP nick end labeling (TUNEL). At the transcript level, changes in gene expression expected to both enhance and inhibit apoptosis were observed in response to increased milking. For instance, pro-apoptotic genes *CIDEA *and *CIDEC *and anti-apoptotic genes *IFI6 *and *PTN *[35] were down regulated, whereas expression of pro-apoptotic genes *ITPR1 *[36], *RAD21 *[37] and *CADM1 *[38] was increased. In addition, expression of *MSR1*, a protein that may be involved in removal of apoptotic cells was increased in IMF4 cows.

Many of the genes that have a role in apoptosis and were down regulated in IMF4 cows were those that participate in ECM turnover and cell adhesion. For instance, expression of the integral membrane proteins *SFRP2 *was down regulated and interaction of this protein with ECM has been shown to prevent apoptosis of mammary epithelial cells [39]. Likewise, *VCAN *and *TIMP1 *were down regulated, and these proteins have been shown to be protective against apoptosis [40-42]. Therefore, the effects of these ECM genes on apoptosis likely are indirect. For instance, loss of cellular contact and signaling with ECM components has been shown to induce apoptosis of endothelial cells by a process termed "vascular pruning" [31]. Further, interaction of mammary epithelial cells with ECM has been shown to be necessary for prevention of cell death by apoptosis [43]. Therefore, it appears that increased apoptosis observed within the mammary epithelial and stromal compartments of IMF4 glands by Hale et al. [5] may be mediated in part by ECM turn over.

Lastly, it is not surprising that changes were observed in the expression of genes related to immune function and inflammation in response to milking frequency. It has been demonstrated that milk SCC, a measure used as a general indicator of mammary infection, increases in the first 24 h in response to IMF [44]. Physical stimulation of the mammary gland and milk removal may also increase the infiltration of immune cells to the gland, as it has been shown that SCC increases substantially from the beginning to the end of each milking period [45]. Thus, variation in presence of immune cells within the gland could be reflected by changes in expression of genes functioning in immunity of cows milked 4× daily versus Controls. In the present study, SCC was measured weekly, but was low in all treatment groups, and no differences in SCC were observed in cows milked 4× daily versus Controls [5].

There is evidence from field studies to suggest that increased frequency of milking of dairy cattle reduces SCC and may be beneficial for herd health [6]. In addition, increased frequency of milk removal from the gland increases the physical removal of bacterial cells from the gland. Therefore, it would be expected that the incidence of mammary infection would be reduced by IMF treatment and that overall expression of genes related to immune function within the mammary gland would be reduced relative to Controls. In the present study, changes in gene expression in IMF4 cows included both down regulation of genes involved in innate immunity, as well as up regulation of acute phase proteins, which appears contradictory. Because gene expression was assessed at approximately d 7 of lactation and approximately 3 d after initiation of 4× daily milking, it is unlikely that changes in health status of the mammary gland due to mammary infection would be evident in such a short time period after the initiation of 4× daily milking. Since the milking process itself increases infiltration of immune cells into the mammary gland, differences in gene expression related to immunity between IMF4 and Control cows were likely influenced by the specific population of cells being sampled during biopsy of the gland, and are therefore difficult to interpret.

## Conclusion

Our data indicate that increased milking frequency in dairy cattle is associated with alteration of mammary cell-ECM interactions and signaling that support milk synthesis. Remodeling of ECM within the gland should enhance mammary epithelial cell proliferation and differentiation, promote apoptosis and cell renewal, and promote migration and invasion of endothelial cells. In addition, increased nutrient delivery to mammary epithelial cells via neovascularization and changes in cell metabolism may enhance secretory capacity of the mammary gland during frequent milking. Increased vascularization of the tissue may also contribute to the persistence of increased milk production after the period of increased milking frequency. Further study is needed to confirm whether the observed changes in gene expression associated with these physiological pathways in fact drive the increases in milk yield during frequent milking, or occur in response to the increase in milk production.

## Authors' contributions

EC conceived and designed the gene expression study, created the SAGE libraries, performed the real-time PCR assays, interpreted results, and wrote the manuscript. SS performed the statistical analysis for the microarray study and contributed to drafting the manuscript. TE conducted the determination of endothelial cell abundance in mammary tissue sections. CC performed the immunohistochemical analyses and contributed to drafting the manuscript. CVT assisted in the evaluation of SAGE tags and their annotation. TS assisted in the creation of the SAGE libraries and their sequence analysis. VF performed the sequence analysis of the SAGE libraries. AC participated in the design of the gene expression study and creation of the SAGE libraries, conceived and designed the immunohistochemistry studies, and directed the original milking frequency experiment by Hale et al. (2003).

## Supplementary Material

Additional file 1 Table. Changes in mammary gene expression in response to four times per day milking (IMF4) as determined by Affymetrix Bovine Genome Array. Click here for file
