# Supplementary material for: Effects of increased milking frequency on gene expression in the bovine mammary gland
Source: BMC Genomics. 2008 Jul 31;9:362. doi: 10.1186/1471-2164-9-362 (PMC2518935; doi:10.1186/1471-2164-9-362)
Supplement: Additional file 1 — Table. Changes in mammary gene expression in response to four times per day milking (IMF4) as determined by Affymetrix Bovine Genome Array. [file 1471-2164-9-362-S1.doc]

| Changes in mammary gene expression in response to four times per day milking^1^ (IMF4) as determined by Affymetrix Bovine Genome Array. | | | | | |
| --- | --- | --- | --- | --- | --- |
| Gene symbol | Fold change | UniGene ID | Gene annotation | Putative function | FDR (%) |
| *ABI3BP* | -1.40 | Bt.31739 | similar to *ABI gene family, member 3 (NESH) binding protein* | unknown | 83.2 |
| *ADIPOQ* | -1.31 | Bt.59365 | *adipocyte complement related protein of 30 kDa* | metabolism | 83.2 |
| *AFF1* | 1.27 | Bt.14634 | similar to *AF-4 (LOC781149)* | transcription factor activity | 56.9 |
| *AGA* | 1.26 | Bt.53989 | similar to *aspartylglucosaminidase* | glycoprotein catabolism | 56.9 |
| *AGTR1* | -1.26 | Bt.4539 | *angiotensin receptor 1 [angiotensin II receptor 1]* | G-protein coupled receptor protein signaling pathway | 83.2 |
| *AKR1C1* | 1.31 | Bt.64294 | *aldo-keto reductase family 1, member C1* | prostaglandin biosynthesis | 83.2 |
| *ALDH5A1* | 1.30 | Bt.2216 | *aldehyde dehydrogenase 5 family, member A1* | mitochondrial electron transport | 83.2 |
| *ANGPTL2* | -1.26 | Bt.21589 | transcribed locus, strongly similar to *angiopoietin-like 2 precursor* [*Homo sapiens*] | induces sprouting of endothelial cells | 83.2 |
| *ANTXR1* | 1.39 | Bt.21981 | similar to *tumor endothelial marker 8 (LOC616010)* | protein binding | 56.9 |
| *AQP5* | 1.26 | Bt.16712 | similar to *aquaporin 5 (LOC782368)* | water transport | 83.2 |
| *ASPN** | 0.10 | Bt.45145 | similar to *asporin precursor (periodontal ligament associated protein 1) (PLAP-1)* | component of proteinaceous extracellular matrix | 83.2 |
| *ASZ1* | -1.25 | Bt.9563 | *ankyrin repeat, SAM and basic leucine zipper domain containing 1* | unknown | 83.2 |
| *ATOX1* | -1.25 | Bt.48850 | similar to *antioxidant protein 1* | copper transport | 83.2 |
| *BACE2* | -1.32 | Bt.53731 | similar to *β-site APP-cleaving enzyme 2 isoform A preproprotein* | proteolysis | 83.2 |
| *BANP* | 2.41 | Bt.11280 | *BTG3 associated nuclear protein* | tumor supressor, cell cycle regulation | 56.9 |
| *BCAS1* | 1.34 | Bt.29499 | similar to *breast carcinoma amplified sequence 1* | putative oncogene | 56.9 |
| *BLA-DQB** | 2.28 | Bt.350 | *MHC class II antigen* | immune response, antigen presentation | 83.2 |
| *BLA-DQB** | 1.51-3.23 | Bt.350 | *MHC class II antigen* | immune response, antigen presentation | 56.9 |
| *BOLA* | -1.48 | Bt.12775 | *classical MHC class I antigen (BOLA)* | antigen presentation | 83.2 |
| *BOLA* | 3.67 | Bt.12775 | *classical MHC class I antigen (BOLA)* | immune response, antigen presentation | 56.9 |
| *BOLA-DQA1** | 2.23 | Bt.4046 | *histocompatibility complex, class II, DQ α, type 1* | immune response, antigen presentation | 83.2 |
| *BoLA-DRB3* | 1.26 | Bt.5356 | *major histocompatibility complex, class II, DRB3* | immune response, antigen presentation | 83.2 |
| *C1orf178* | 1.49 | Bt.47926 | similar to *pro-apoptotic Bcl-2 protein (Bfk)* | apoptosis, mammary development | 83.2 |
| *CADM1* | 1.26 | Bt.89712 | *cell adhesion molecule 1* | positive regulation of cytokine secretion, cell adhesion | 56.9 |
| *CBX5* | -1.26 | Bt.52233 | *chromobox homolog 5 (HP1 α homolog,* *Drosophila*) | chromatin assembly and disassembly | 83.2 |
| *CCL26* | -1.30 | Bt.23451 | similar to *chemokine (C-C motif) ligand 26 precursor* | immune response | 83.2 |
| *CFB* | -1.34 | Bt.13542 | similar to *complement factor B precursor (C3/C5 convertase)* | immune response, complement activation | 83.2 |
| *CHRDL1* | -1.39 | Bt.3213 | similar to *ventroptin* | cell differentiation | 83.2 |
| *CIDEA* | -1.32 | Bt.20167 | similar to *cell death-inducing DFFA-like effector a isoform 2* | activator of apoptosis | 83.2 |
| *CIDEC* | -1.51 | Bt.13381 | similar to *cell death activator CIDE-3* | induction of apoptosis | 83.2 |
| *CILP* | -1.40 | Bt.13884 | similar to *cartilage intermediate layer protein, nucleotide pyrophosphohydrolase* | component of proteinaceous extracellular matrix | 83.2 |
| *COL1A1** | 0.13 | Bt.23316 | *collagen, type I, α 1* | extracellular matrix structural constituent | 83.2 |
| *COL1A2** | 0.10 | Bt.53485 | *collagen, type I, α 2* | extracellular matrix structural constituent | 83.2 |
| *COL3A1** | 0.08 | Bt.64714 | similar to *collagen α 1(III) chain precursor* | extracellular matrix structural constituent | 83.2 |
| *COL5A2* | -1.26 | Bt.55266 | similar to *procollagen, type V, α 2* | extracellular matrix structural component | 83.2 |
| *COL6A1* | -1.28 | Bt.23508 | similar to *collagen α 1(VI) chain precursor* | extracellular matrix structural constituent | 83.2 |
| *COL6A3* | -1.72 | Bt.68159 | similar to *α 3 type VI collagen isoform 3 precursor* | cell adhesion | 83.2 |
| *COL12A1** | 0.06 | Bt.7380 | *collagen, type XII, α 1* | extracellular matrix structural constituent | 83.2 |
| *COX7C* | -1.25 | Bt.77 | *cytochrome c oxidase subunit VIIc* | electron transfer in mitochondrion | 83.2 |
| *CPT1A* | 1.31 | Bt.9289 | similar to *carnitine O-palmitoyltransferase I* | fatty acid beta oxidation and transport | 83.2 |
| *CROT* | 1.27 | Bt.65661 | *carnitine O-octanoyltransferase* | fatty acid transport | 83.2 |
| *CRYAB* | 1.34 | Bt.88059 | *crystallin, α polypeptide 2* | transmembrane receptor protein tyrosine kinase signaling | 83.2 |
| *CSTB* | -1.46 | Bt.36484 | similar to *cystatin B (liver thiol proteinase inhibitor) (CPI-B) (stefin B)* | edopeptidase inhibitor activity | 83.2 |
| *CTGF* | 1.35 | Bt.5240 | *connective tissue growth factor* | cell adhesion and regulation of cell growth | 83.2 |
| *CTSK* | -1.31 | Bt.23218 | *cathepsin K preproprotein* | proteolysis | 83.2 |
| *CXCL14* | 1.42 | Bt.20397 | similar to *small inducible cytokine B14 precursor* | homeostasis of monocyte-derived macrophages | 83.2 |
| *CXCR7* | -1.44 | Bt.27379 | similar to *chemokine orphan receptor 1* | G protein coupled receptor activity | 83.2 |
| *DAB2* | -1.25 | Bt.15382 | similar to *disabled homolog 2 (differentially expressed protein 2) (DOC-2)* | cell proliferation, putative tumor suppressor | 83.2 |
| *DIRAS3* | 1.27 | Bt.49617 | *DIRAS family, GTP-binding RAS-like 3* | regulation of cyclin-dependent protein kinase activity, putative tumor suppressor | 56.9 |
| *DKFZP564O0823* | 1.29 | Bt.13546 | similar to *DKFZP564O0823 protein* | unknown | 56.9 |
| *DPT* | -1.50 | Bt.40988 | similar to *dermatopontin precursor (tyrosine-rich acidic matrix protein) (TRAMP)* | cell adhesion | 83.2 |
| *ECM1* | -1.38 | Bt.5525 | similar to *extracellular matrix protein 1 isoform 1 precursor* | positive regulation of I-κB kinase/NF-κB cascade, cell differentiation | 83.2 |
| *EFEMP1* | -1.31 | Bt.40413 | similar to *EGF-containing fibulin-like extracellular matrix protein 1 isoform b* | calcium ion binding, component of proteinaceous extracellular matrix | 83.2 |
| *EGR1* | -1.33 | Bt.54463 | *early growth response protein 1* | regulation of transcription | 83.2 |
| *ELN* | -1.33 | Bt.5361 | *elastin* | cell proliferation | 83.2 |
| *ENTPD4* | 1.25 | Bt.29990 | similar to *ectonucleoside triphosphate diphosphohydrolase 4* | UDP catabolic process | 56.9 |
| *F13A1* | -1.26 | Bt.19195 | similar to *coagulation factor XIII A chain precursor* | blood coagulation | 83.2 |
| *F2R** | 0.02 | Bt.10814 | similar to *coagulation factor II (thrombin) receptor* | thrombotic response, angiogenesis | 83.2 |
| *FAP* | -1.47 | Bt.2717 | similar to *fibroblast activation protein, α subunit* | proteolysis | 83.2 |
| *FAT2* | 1.29 | Bt.10136 | similar to *FAT tumor suppressor 2 precursor* | regulation of cell proliferation | 56.9 |
| *FBLN1* | -1.36 | Bt.61392 | similar to *fibulin 1 isoform C precursor* | extracellular matrix structural component | 83.2 |
| *FBN1* | -1.46 | Bt.5021 | *fibrillin 1* | extracellular matrix structural component | 83.2 |
| *FGFR2* | 1.26 | Bt.8088 | *fibroblast growth factor receptor 2* | positive regulation of epithelial cell proliferation | 56.9 |
| *FGG* | 1.25 | Bt.48905 | *fibrinogen, γ polypeptide* | inflammatory response, positive regulation of cell proliferation | 83.2 |
| *FGL2* | -1.30 | Bt.39517 | *fibrinogen-like 2* | link between proinflammatory cytokines and generation of active thrombin | 83.2 |
| *FHL1* | -1.30 | Bt.23401 | similar to *four and a half LIM domains protein 1* | cell growth and differentiation | 83.2 |
| *FN1* | -1.60 | Bt.23418 | *fibronectin 1* | acute phase response, cell adhesion | 83.2 |
| *FSTL1* | -1.32 | Bt.40414 | *follistatin-like 1* | heparin binding, calcium ion binding | 83.2 |
| *G0S2* | -1.26 | Bt.4137 | similar to *putative lymphocyte G0/G1 switch protein 2* | regulation of cell cycle, adipocyte differentiation | 83.2 |
| *GPNMB* | -1.28 | Bt.9807 | *glycoprotein (transmembrane) nmb* | negative regulation of cell proliferation | 83.2 |
| *GPX3* | -1.27 | Bt.12916 | *glutathione peroxidase 3 (plasma)* | protection of cells against oxidative damage | 83.2 |
| *GSN* | -1.36 | Bt.53493 | similar to *gelsolin precursor (actin-depolymerizing factor)* | component of actin cytoskeleton | 83.2 |
| *HF1** | 1.27-1.30 | Bt.33303 | similar to *complement factor H precursor (H factor 1)* | complement activation | 83.2 |
| *HRASLS3* | 1.31 | Bt.13534 | similar to *HRAS* *like suppressor 3* | negative regulation of progression through cell cycle | 83.2 |
| *HSPB6* | -1.28 | Bt.7478 | similar to heat *shock protein, α-crystallin-related, B6* | protein binding | 83.2 |
| *HSPB8* | -1.26 | Bt.22526 | *heat shock 27kDa protein 8* | protein folding | 83.2 |
| *IDH1* | -1.26 | Bt.13324 | *isocitrate dehydrogenase 1 (NADP+), soluble* | TCA cycle | 83.2 |
| *IDI1* | 1.27 | Bt.3764 | similar to *isopentenyl-diphosphate δ isomerase* | isoprenoid biosynthesis | 56.9 |
| *IFI6* | -1.26 | Bt.8436 | *interferon, α-inducible protein 6* | inhibitor of apoptosis | 83.2 |
| *IGFBP6* | -1.38 | Bt.9958 | *insulin-like growth factor-binding protein 6* | negative regulation of cell proliferation, regulation of cell growth | 83.2 |
| *IGHA1* | -1.50 | Bt.8875 | *immunoglobulin heavy constant α 1* | protein binding | 83.2 |
| *IL1RAP* | 1.25 | Bt.28708 | similar to *interleukin-1 receptor accessory protein precursor* | immune response, inflammatory response | 56.9 |
| *ISG12(A)* | -1.32 | Bt.46545 | similar to *putative ISG12(a) protein* | response to pest, pathogen or parasite | 83.2 |
| *ITPR1* | 1.43 | Bt.22305 | *inositol 1,4,5-triphosphate receptor, type 1* | inositol-1,4,5-triphosphate receptor activity | 83.2 |
| *KIAA1199* | -1.53 | Bt.44380 | transcribed locus | unknown | 83.2 |
| *KLF11** | 1.26-1.33 | Bt.16916 | *Kruppel-like factor 11* | negative regulation of cell proliferation | 56.9-83.2 |
| *KRT18* | -1.33 | Bt.65129 | similar to *keratin, type I cytoskeletal 18* | structural constituent of cytoskeleton | 83.2 |
| *LOC404103* | -1.39 | Bt.28518 | *spleen trypsin inhibitor* | endopeptidase inhibitor activity | 83.2 |
| *LOC493778* | 1.37 | Bt.837 | *up-regulated during vascular calcification* | unknown | 56.9 |
| *LOC504773* | -1.26 | Bt.6556 | *regakine-1 protein* | immune response, inflammatory chemokine | 83.2 |
| *LOC510417* | -1.37 | Bt.60905 | *MHC class I heavy chain* | antigen presentation | 83.2 |
| *LOC504548* | -1.26 | Bt.62650 | similar to *LOC512938 protein* | unknown | 83.2 |
| *LOC515340* | -1.25 | Bt.29579 | *N-acetylgalactosaminyltransferase A blood group-like* | carbohydrate metabolism | 83.2 |
| *LOC521730* | 1.26 | Bt.64905 | similar to *elongation factor-2 kinase* | unknown | 56.9 |
| *LOC616204* | 1.26 | Bt.11772 | transcribed locus | unknown | 56.9 |
| *LOC617040* | -1.27 | Bt.1372 | *hypothetical protein LOC617040* | unknown | 83.2 |
| *LOC785366* | 1.66 | Bt.18855 | similar to *embigin homolog* | unknown | 56.9 |
| *LOC787253* | -1.25 | Bt.9675 | similar to *extracellular proteinase inhibitor* | unknown | 83.2 |
| *LOC790811* | 1.33 | Bt.28022 | similar to *MHC class I antigen* | unknown | 56.9 |
| *LOX* | -1.34 | Bt.36642 | *lysyl oxidase* | protein tyrosine kinase signalling pathway, putative tumor supressor | 83.2 |
| *LUM* | -1.43 | Bt.2452 | *lumican* | collagen fibril organization | 83.2 |
| *MFAP2* | -1.26 | Bt.22115 | *microfibrillar-associated protein 2* | extracellular matrix structural component | 83.2 |
| *MFAP5* | -1.55 | Bt.2560 | *microfibrillar associated protein 5* | component of proteinaceous extracellular matrix | 83.2 |
| *MGC127724* | -1.27 | Bt.49302 | *6.8 kDa mitochondrial proteolipid* | mitochondrial proteolipid | 83.2 |
| *MGP* | -1.25 | Bt.3595 | *matrix Gla protein* | ossification, calcium ion binding | 83.2 |
| *MIA* | 1.31 | Bt.4267 | *melanoma inhibitory activity* | cell proliferation | 56.9 |
| *mitochondrial RNA* | -1.50 | --- | *mitochondrial RNA, similar to 18S rRNA* | unknown | 83.2 |
| *MSR1* | 1.27 | Bt.4482 | *macrophage scavenger receptor 1* | receptor-mediated endocytosis | 83.2 |
| *MUC15* | -1.26 | Bt.11217 | *mucin 15* | component of milk fat globular membrane | 83.2 |
| *MUSTN1* | -1.26 | Bt.9832 | transcribed locus, strongly similar to *musculoskeletal*, *embryonic nuclear protein 1 [Homo sapiens]* | unknown | 83.2 |
| *MYBPC1* | -1.37 | Bt.25860 | similar to *myosin binding protein C, slow type isoform 3* | cell adhesion, structural constituent of muscle | 83.2 |
| *MYOC* | -1.33 | Bt.4703 | *myocilin, trabecular meshwork inducible glucocorticoid response* | structural molecule activity | 83.2 |
| *NDRG2* | 1.28 | Bt.49532 | *NDRG family member 2* | cell differentiation | 56.9 |
| *NELL2* | -1.43 | Bt.8804 | similar to *protein kinase C-binding protein NELL2 precursor (NEL-like protein 2)* | regulation of cell growth and differentiation, cell adhesion | 83.2 |
| *NOV** | 0.25 | Bt.27716 | similar to *NOV protein homolog precursor* | regulation of cell growth | 83.2 |
| *NRG1* | 1.28 | Bt.411 | *neuregulin 1* | induces cell growth and differentiation | 56.9 |
| *NT5E** | 0.10 | Bt.57034 | *5'-nucleotidase, ecto (CD73)* | nucleotide catabolism | 83.2 |
| *NTRK2* | -1.27 | Bt.64757 | *neurotrophic tyrosine kinase, receptor, type 2* | cell differentiation | 83.2 |
| *OGN* | -1.39 | Bt.5341 | *osteoglycin (osteoinductive factor, mimecan)* | growth factor activity | 83.2 |
| *OLFML3* | -1.29 | Bt.23129 | similar to *olfactomedin-like protein 3 precursor (HNOEL-iso)* | cellular component | 83.2 |
| *PCOLCE** | -1.29 | Bt.7955 | similar to *procollagen C-endopeptidase enhancer* | collagen binding, proteolysis | 83.2 |
| *PDGFC* | 1.25 | Bt.3014 | similar to *platelet-derived growth factor C precursor* | positive regulation of cell proliferation | 56.9 |
| *PDGFRL* | -1.33 | Bt.47512 | *platelet-derived growth factor receptor-like protein* | putative tumor suppressor | 83.2 |
| *PDLIM3* | 1.27 | Bt.57571 | *PDZ and LIM domain 3* | actin filament organization | 56.9 |
| *PHLDA1* | 1.26 | Bt.56381 | *Pleckstrin homology-like domain, family A, member 1* | role in anti-apoptotic effects of IGF-I | 56.9 |
| *PI16* | -1.49 | Bt.4912 | *protease inhibitor 16* | peptidase activity | 83.2 |
| *PIGR* | -1.36 | Bt.4695 | *polymeric immunoglobulin receptor* | transport of IgA, IgM into mammary secretions | 83.2 |
| *PIK3R1* | 1.32 | Bt.56561 | *phosphoinositide-3-kinase, regulatory subunit 1 (p85 α)* | insulin-like growth factor receptor signaling, negative regulation of apoptosis and cell adhesion | 56.9 |
| *PMP22* | -1.47 | Bt.22534 | similar to *peripheral myelin protein 22* | negative regulation of cell proliferation, cell differentiation | 83.2 |
| *POLR2L* | -1.28 | Bt.66354 | *polymerase (RNA) II (DNA directed) polypeptide L, 7.6kDa* | regulation of transcription | 83.2 |
| *PTN* | -1.33 | Bt.4844 | *pleiotrophin* | positive regulation of cell proliferation | 83.2 |
| *RAD21* | 1.28 | Bt.15668 | *RAD21 homolog (S. pombe)* | repair of DNA double-strand breaks during mitosis | 56.9 |
| *RARRES1* | -1.50 | Bt.53829 | similar to *retinoic acid receptor responder (tazarotene induced) 1 isoform 1* | negative regulation of cell proliferation | 83.2 |
| *RASL11B* | -1.39 | Bt.1974 | *RAS-like, family 11, member B* | negative regulation of cell growth | 83.2 |
| *RASSF4** | 0.01 | Bt.45219 | similar to *ras association domain family 4 isoform a* | negative regulation of cell cycle, putative tumor suppressor | 83.2 |
| *RGS2* | 1.41 | Bt.63864 | similar to *regulator of G-protein signalling 2, 24kDa* | negative regulation of G-protein coupled receptor protein signaling pathway | 56.9 |
| *RPL37A* | -1.26 | Bt.16064 | *60S ribosomal protein L37A* | protein biosynthesis | 83.2 |
| *RPL38* | -1.27 | Bt.7658 | similar to *60S ribosomal protein L38* | translation | 83.2 |
| *RPS21* | -1.31 | Bt.7391 | *ribosomal protein S21* | structural constituent of ribosome, cell proliferation | 83.2 |
| *RPS28* | -1.31 | Bt.22772 | *ribosomal protein S28* | protein biosynthesis | 83.2 |
| *S100A10* | -1.37 | Bt.5037 | *S100 calcium-binding protein A10* | regulation of cell growth and differentiation | 83.2 |
| *S100A4* | -1.57 | Bt.49733 | *S100 calcium binding protein A4* | regulation of cell cycle and differentiation | 83.2 |
| *SC4MOL* | 1.25 | Bt.23212 | similar to *C-4 methylsterol oxidase* | cholesterol biosynthesis, fatty acid metabolism | 56.9 |
| *SCARA5* | -1.25 | Bt.76787 | *scavenger receptor class A, member 5* | phosphate transport | 83.2 |
| *SCUBE2* | -1.28 | Bt.25478 | similar to *CEGP1 protein* | development, inflammation | 83.2 |
| *SERPINF1* | -1.57 | Bt.2638 | *serine (or cysteine) proteinase inhibitor, clade F, member 1* | cell proliferation | 83.2 |
| *SERPING1* | -1.51 | Bt.22969 | *serine (or cysteine) proteinase inhibitor, clade G (C1 inhibitor), member 1* | negative regulation of complement activation, classical pathway | 83.2 |
| *SFRP2* | -1.38 | Bt.40491 | *secreted frizzled-related protein 2* | modulator of Wnt signaling | 83.2 |
| *SFRP4* | -1.88 | Bt.3540 | similar to *secreted frizzled-related protein 4 precursor* | modulator of Wnt signaling, transcription | 83.2 |
| *SLC1A5* | 1.51 | Bt.5548 | *solute carrier family 1 (neutral amino acid transporter), member 5* | neutral amino acid transport | 56.9 |
| *SLC34A2* | -1.31 | Bt.61466 | *solute carrier family 34 (sodium phosphate), member 2* | ion transport | 83.2 |
| *SLC39A8* | 1.36 | Bt.22697 | similar to *solute carrier family 39 (zinc transporter), member 8* | metal ion transport | 56.9 |
| *SLC7A5* | 1.27 | Bt.5528 | *solute carrier family 7 (cationic amino acid transporter, y+ system), member 5* | amino acid transport | 83.2 |
| *SOX9* | 1.27 | Bt.30086 | moderately similar to *transcription factor SOX-9* | transcription factor activity | 56.9 |
| *SPADH1* | 2.27 | Bt.457 | *spermadhesin 1* | growth factor activity | 56.9 |
| *SPARCL1* | -1.41 | Bt.8880 | similar to *SPARC-like protein 1 precursor (high endothelial venule protein)* | calcium ion binding, component of proteinaceous extracellular matrix | 83.2 |
| *SYT17* | 1.42 | Bt.4414 | similar to *B/K protein* | transporter activity | 56.9 |
| *TCF7L2* | 1.27 | Bt.43658 | similar to *transcription factor 7-like 2, T-cell-specific, HMG-box* | regulation of progression through cell cycle | 56.9 |
| *TGFBI* | -1.25 | Bt.46833 | *transforming growth factor-β induced protein IG-H3 precursor (β IG-H3)* | negative regulation of cell adhesion, cell proliferation | 83.2 |
| *THBS2* | -1.33 | Bt.5522 | *thrombospondin 2* | cell adhesion | 83.2 |
| *THBS4* | -1.38 | Bt.11641 | similar to *thrombospondin 4 precursor* | cell adhesion | 83.2 |
| *THOC4* | 1.26 | Bt.36494 | similar to *signal-induced proliferation-associated 1 like 1* | mRNA processing | 56.9 |
| *THRAP3* | -1.46 | Bt.45504 | similar to *thyroid hormone receptor associated protein 3* | positive regulation of transcription | 83.2 |
| *TIMP1** | 0.08 | Bt.27976 | *tissue inhibitor of metalloproteinase 1 (erythroid potentiating activity, collagenase inhibitor)* | positive regulation of cell proliferation, negative regulation of apoptosis | 83.2 |
| *TIMP2** | -1.28 | Bt.52974 | *tissue inhibitor of metalloproteinase 2* | negative regulation of cell proliferation | 83.2 |
| *TMED10* | 1.25 | Bt.87833 | *transmembrane trafficking protein* | vesicular protein trafficking | 56.9 |
| *TNC* | -1.45 | Bt.11061 | similar to *tenascin C* | negative regulation of cell adhesion | 83.2 |
| *TNXB* | -1.25 | Bt.5398 | *tenascin X* | negative regulation of cell adhesion, extracellular matrix structural constituent | 83.2 |
| *TSC22D1* | 1.26 | Bt.10953 | similar to *transforming growth factor β 1 induced transcript 4 protein* | regulation of transcription | 83.2 |
| *UBD* | -1.33 | Bt.49039 | similar *to ubiquitin D* | anitmicrobial humoral response | 83.2 |
| *VCAN** | 0.09 | Bt.5395 | *versican* | cell adhesion | 83.2 |
| --- | -1.35 | Bt.29581 | transcribed locus | unknown | 83.2 |
| --- | -1.35 | Bt.3202 | transcribed locus | unknown | 83.2 |
| --- | -1.31 | Bt.19295 | transcribed locus | unknown | 83.2 |
| --- | -1.29 | Bt.23342 | transcribed locus | unknown | 83.2 |
| --- | 1.25 | Bt.90043 | transcribed locus | unknown | 56.9 |
| --- | 1.26 | Bt.89717 | transcribed locus | unknown | 56.9 |
| --- | 1.26 | Bt.20074 | transcribed locus | unknown | 56.9 |
| --- | 1.26 | Bt.5519 | transcribed locus | unknown | 56.9 |
| --- | 1.29 | Bt.55680 | transcribed locus | unknown | 56.9 |
| --- | 1.37 | Bt.21688 | transcribed locus | unknown | 83.2 |
| --- | 1.38 | Bt.20162 | transcribed locus | unknown | 56.9 |
| --- | 1.39 | Bt.640 | transcribed locus | unknown | 83.2 |
| --- | 1.72 | Bt.22729 | transcribed locus | unknown | 56.9 |
| --- | 1.94 | Bt.65714 | transcribed locus | unknown | 56.9 |
| --- | 2.06 | Bt.27292 | transcribed locus | unknown | 56.9 |

^1^ Cows were milked twice daily from d 1 to 3 of lactation, and four times daily beginning at d 4 of lactation until d 21 post partum

* Differential expression detected by more than one probe ID.
